# Basal Ganglia Calcification: A Case Report of Two Siblings With Fahr's Disease

**DOI:** 10.7759/cureus.53434

**Published:** 2024-02-02

**Authors:** Margarida Magalhães, Margarida Alves, Luís Paulino Ferreira, Janice Alves, Diana Durães

**Affiliations:** 1 Department of Psychiatry and Mental Health, Setúbal Hospital Centre, Setúbal, PRT; 2 Department of Neurosciences, Nova Medical School, Lisbon, PRT; 3 Department of Neurology, Setúbal Hospital Centre, Setúbal, PRT

**Keywords:** computed tomography, case report, neuropsychiatry, basal ganglia calcification, fahr's disease

## Abstract

Fahr's disease is a rare neurodegenerative disorder caused by bilateral and usually symmetrical intracranial calcifications. In most cases, it exhibits an autosomal dominant pattern of inheritance and genetic heterogeneity. Patients may present with movement disorders, cognitive impairment, and psychiatric disorders. Currently, there are no disease-modifying drugs, so the management is based on the treatment of the symptoms. We present two cases involving male siblings, both with psychiatric symptoms as the initial presentation of the disease. Brain computed tomography revealed bilateral calcifications in the basal ganglia for which no underlying cause was found. In both cases, remission of behavioural changes and psychiatric symptoms was achieved with psychotropic drugs.

## Introduction

Fahr's disease (FD), also known as idiopathic basal ganglia calcification, was first described by Karl T. Fahr in 1930. It is a rare neurodegenerative disorder caused by bilateral and usually symmetrical basal ganglia calcifications, although it may extend to other regions as well [1]. According to recent data, it has an estimated prevalence of 4.5 per 10,000 persons [2]. It commonly exhibits an autosomal dominant inheritance, and seven genes have been implicated in its genetic aetiology, of which four with dominant inheritance (SLC20A2, PDGFB, PDGFRB, XPR1) and three with recessive inheritance (MYORG, JAM2, CMPK2) [1,3]. The mean age of clinical onset is around 40-50 years, but symptoms can manifest at any age [4]. Patients with FD may be asymptomatic [1]. However, they can present with cognitive impairment, movement disorders (for example, parkinsonism), and psychiatric disorders such as anxiety, depression, or psychotic symptoms [1]. This clinical heterogeneity can be partly explained by the basal ganglia's division into dorsal and ventral systems. The dorsal striatum is associated with motor and cognitive functions, whereas the ventral striatum is responsible for motivational functions [1]. Calcified areas are identified as hyperdense lesions on unenhanced brain computed tomography (CT), which is the most reliable exam for the diagnosis. These calcifications are commonly symmetrical, most frequently found in the basal ganglia, but they may also involve the cerebellar dentate nuclei, thalamus, cerebral cortex, subcortical white matter, and hippocampus [3,5].

We present two cases of siblings, one aged 46 (case 1) and the other aged 42 (case 2), both with psychiatric symptoms as the initial presentation of FD. This paper will also discuss the neuropathological aspects, clinical manifestations, features related to evaluation and diagnosis, and management of FD/syndrome.

## Case presentation

Case 1 

Mr. S, a 46-year-old Angolan man, presented to the emergency room (ER) with complaints of behavioural alterations, including aggressiveness, psychomotor agitation, and suicidal ideation. According to his past medical history, he had normal psychomotor development until he was about one and a half years of age when he presented with a loss of some acquired skills (walking and talking). Later, he exhibited severe cognitive impairment. In school, he struggled to learn how to read or write, perform simple calculations, or understand the value of money. Throughout his life, he needed help to perform daily activities such as personal hygiene and feeding, remaining unable to become independent. 

In his 40s, he began experiencing episodes of psychomotor agitation and aggressiveness and was prescribed risperidone 1 mg (once per day), mirtazapine 15 mg (once per day), and clonazepam 0.5 mg (once per day), leading to the remission of symptoms. However, a few months prior to the ER visit, he had discontinued all medication, which led to the behavioural alterations previously described. He was referred to a psychiatry consultation and restarted his medication.

Laboratory investigations revealed mild pancytopenia. Other laboratory findings, including urinalysis, fasting blood glucose, blood electrolytes, phosphate, creatine phosphokinase, vitamin D, thyroid hormones, hepatic enzymes, and iron and transferrin levels, showed no abnormalities. Blood calcium was 8.8 mg/dl (normal range: 8.4-10.2 mg/dl), and parathyroid hormone levels were 54.1 pg/ml (normal range: 15-68.3 pg/ml), all within normal limits. There was no laboratory evidence of infectious diseases, including infection with human immunodeficiency viruses 1 and 2, hepatitis B and C, and syphilis. 

**Figure 1:**
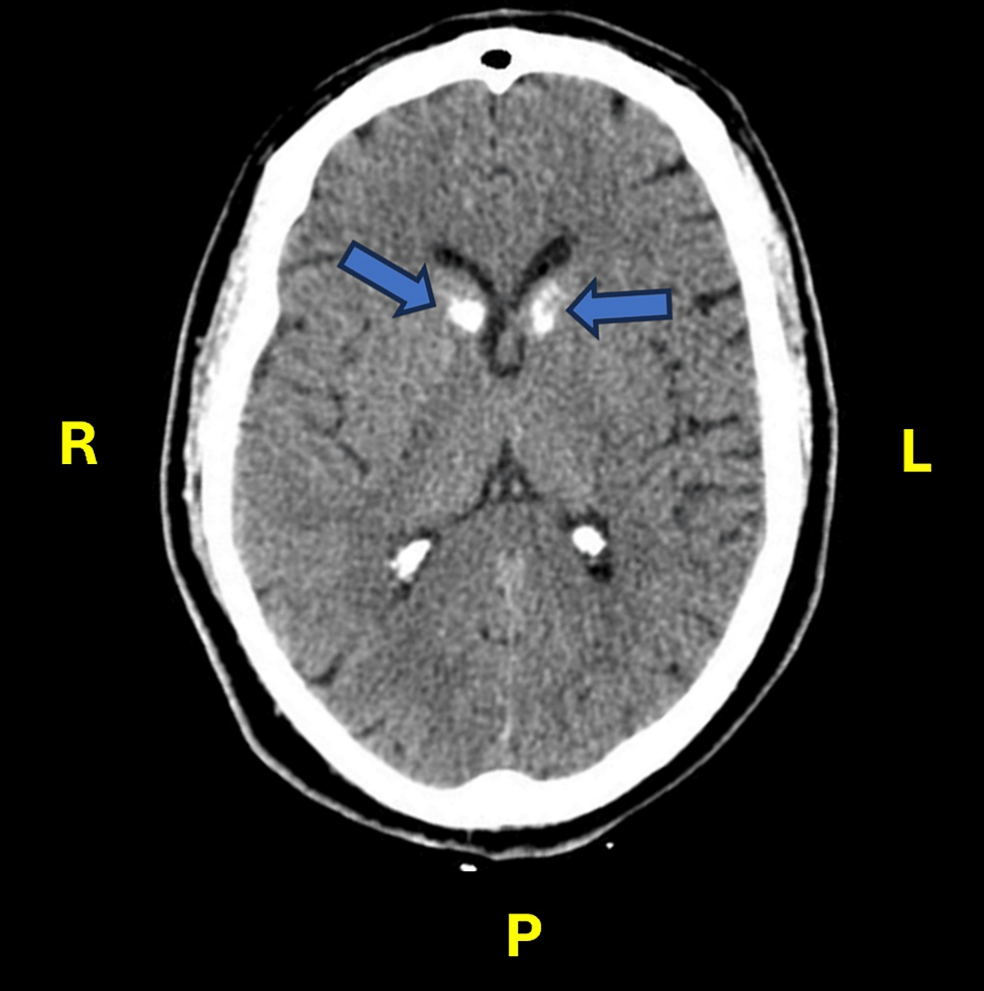
Brain CT scan image (axial section) of case 1 showing bilateral calcifications in the basal nuclei (blue arrows). CT: computed tomography

A brain CT scan revealed bilateral calcifications in the basal ganglia involving the head of the caudate nucleus and anterior lenticular regions, indicated by high-density white areas, as illustrated in Figure 1. The electroencephalogram (EEG) showed a general slowing of brain activity with no paroxysmal activity.

A neuropsychological assessment was conducted (intelligence quotient was not quantified), revealing limited cognitive-operative plasticity suggestive of impairment in the frontal-limbic pathways, which translates into a severe intellectual developmental disorder, particularly affecting the conceptual, social, and practical domains.

During the examination, he was vigilant and cooperative within his means. However, there was disorientation regarding place and time. Speech was dysarthric, sparse, and only provoked; at times, words were incomprehensible. Thought capacity was poor, with marked psychomotor lentification. The mood was dysphoric. There were no delusional ideas or hallucinations. Physical examination also revealed walking impairment, characterized by a wide-based gait and imbalance. There was no evidence suggestive of bipolar disorder.

On evaluation during psychiatric consultation, after resuming previous medications, the patient showed a positive response, with the remission of behavioural alterations and suicidal ideation. However, he continued to experience the aforementioned cognitive and gait impairment.

Case 2

Mr. T, a 42-year-old Angolan man, was referred to a psychiatric consultation by his family doctor due to anxiety and depressive mood, without psychotic symptoms. According to his past medical history, he experienced normal psychomotor development until the age of nine months when he suffered meningoencephalitis caused by the measles virus. Subsequently, he began to exhibit delayed cognitive development and generalized tonic-clonic seizures. However, he has been seizure-free since adolescence and has not required antiepileptic medication. He faced learning difficulties in school and was never able to get a job. Also, he is partially dependent on others for everyday activities, such as cooking meals.

During the psychiatric observation, he was vigilant, calm, and collaborative, with a depressive and anxious mood. No delusional ideas or hallucinations were observed. There was no evidence of focal or pyramidal deficits, changes in coordination, or alterations in cranial nerves or gait. There was no evidence suggestive of bipolar disorder.

Laboratory findings, including urinalysis, blood count, fasting blood glucose, blood electrolytes, vitamin D, thyroid hormones, phosphate, creatine phosphokinase, hepatic enzymes, and iron and transferrin levels, showed no abnormalities. Blood calcium was 9.7 mg/dl (normal range: 8.4-10.2 mg/dl), and parathyroid hormone levels were 61.3 pg/ml (normal range: 15-68.3 pg/ml). There was no laboratory evidence of infectious diseases, including infection with human immunodeficiency viruses 1 and 2, hepatitis B and C, and syphilis. 

**Figure 2:**
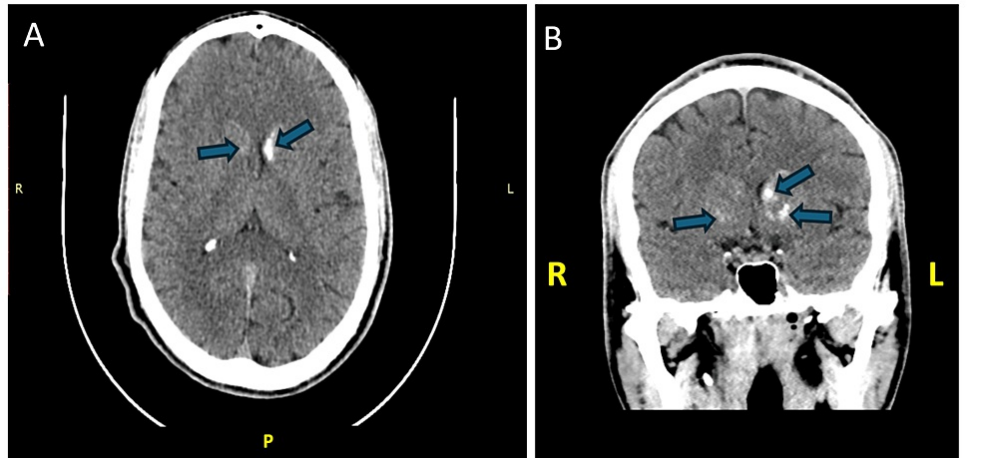
Brain CT scan images (A, axial section; B, coronal section) of case 2 showing bilateral calcifications in the caudate nucleus and lenticular nuclei (blue arrows). CT: computed tomography

A brain CT scan (Figure 2) revealed calcifications in the head of the caudate nucleus, as well as in the lenticular nuclei bilaterally and adjacent to Monroe's foramina, with a more pronounced presence on the left side. Additionally, calcifications were identified next to the wall of the left lateral ventricle, particularly near the left frontal horn. The EEG showed slightly slow baseline electrogenesis with no paroxysmal activity.

He was prescribed escitalopram 10 mg per day and gabapentin 100 mg three times a day, resulting in a complete symptomatic response.

## Discussion

FD is predominantly autosomal dominantly inherited, and mutations have been identified in four causative genes (SLC20A2, XPR1, PDGFB, and PDGFRB) [1]. Nevertheless, around 50% of FD patients have yet to receive a genetic diagnosis [6]. The physiopathological mechanism of FD is not completely understood. Anatomopathological alterations were mainly observed in small vessels, perivascular regions, neuroglia, and neurons [7].

However, one must distinguish FD from Fahr's syndrome. FD is idiopathic or genetic. In Fahr's syndrome, the basal ganglia calcifications are secondary to an underlying systemic disease, such as parathyroid abnormalities, mitochondrial myopathies, and infections. FD must also be distinguished from "radiological" basal ganglia calcification without clinical features, which is found on CT scans in 0.9% of the general population [8].

The available epidemiological data suggests that this disease is more common in men [9], with clinical onset typically occurring around 40-50 years of age. Clinical manifestations can range from asymptomatic cases to more severe courses, even among individuals within the same family [10]. Severe forms may lead to progressive deterioration, encompassing psychosis, depression, dementia, gait disturbance, speech difficulties, basal ganglia movement disorders, and cerebellar signs [5,10,11].

Motor manifestations were reported in approximately one-third of patients [3]. About 42% of cases display anxiety, psychosis, cognitive decline, and headache [3]. Parkinsonism represents the primary motor phenotype in FD [12].

The diagnosis of FD relies on the identification of bilateral basal ganglia calcifications through brain imaging (CT scan) and the exclusion of secondary causes of calcium deposition in the brain [3]. These secondary causes encompass persistent hypocalcemia resulting from calcium metabolism alterations, infectious diseases, as well as other rare neurodegenerative conditions [3]. 

The diagnostic criteria for FD, originally derived from Moskowitz et al., have undergone modifications and can be outlined as follows [13,14]: (a) bilateral calcification of the basal ganglia or other brain regions; (b) progressive neurologic dysfunction, most often a movement disorder and/or neuropsychiatric manifestations; (c) onset typically occurs in the fourth or fifth decade; (d) absence of a mitochondrial or metabolic disease or other systemic disorder; (e) absence of an infectious, toxic, or traumatic cause; and (f) family history consistent with autosomal dominant inheritance.

In our case report, all the diagnostic criteria were met for case 1, and five of the diagnostic criteria were met for case 2. Laboratory screening should include a complete calcium metabolism assessment, lactic acid, and creatine phosphokinase (potentially increased in mitochondrial diseases) [11]. In individuals diagnosed with FD, serum levels of calcium, phosphate, alkaline phosphatase, and parathyroid hormone are within normal ranges [1]. Concerning imaging tests, a brain CT scan is the most sensitive modality for localizing and assessing the extent of calcium deposits. The lenticular nucleus, particularly the internal globus pallidus, is the most affected area. A fluorodeoxyglucose-positron emission tomography (FDG-PET) may also be required and is expected to show decreased FDG uptake, especially in the basal ganglia [11].

The calcifications appear to follow a typical progression, starting with mild calcification limited to the lentiform nucleus and caudate nucleus and advancing to severe calcification affecting all basal ganglia except the midbrain [4]. The severity of calcifications on CT is positively correlated with age, and symptomatic patients exhibit more calcifications compared to asymptomatic patients [2]. Radiological follow-up has limited value in clinical practice. This is because it is not possible to establish the disease prognosis based on radiological findings, even in cases where the progression of calcification on brain imaging is demonstrable over time [3].

Our cases, both males and brothers, have experienced some degree of cognitive and motor impairment since childhood. Case 2 has a history of childhood infection, while Case 1 has no history of infection or traumatic events. They presented with neuropsychiatric symptoms (anxiety, depression, suicidal crisis) starting in the fifth decade of life, revealing significant calcifications in the basal ganglia during neuroimaging assessment. Blood tests showed no significant abnormalities: blood count, renal function tests, parathyroid hormone, thyroid hormones, and vitamin D were all normal. In our cases, five of the diagnostic criteria were met. The absence of laboratory findings, EEG changes, and somatic characteristics suggestive of mitochondrial, metabolic, or other systemic diseases reinforces the diagnosis of FD.

Currently, there is no disease-modifying therapy for FD to reduce brain calcification or prevent its progression; thus, the treatment is mainly symptomatic [6]. Due to the large clinical heterogeneity, various medications are employed, including antipsychotics, anticonvulsants, antidepressants, mood stabilizers, antiparkinsonian drugs, analgesics, and benzodiazepines [15].

Selective serotonin reuptake inhibitors (SSRIs) are employed for managing depression, obsessive-compulsive behaviours, and anxiety in FD. Caution is warranted when using carbamazepine and barbiturates, as they may exacerbate gait dysfunction [11]. Psychiatrists and neurologists should exercise great caution with the use of antidepressants and anxiolytics, as these drugs can have a low threshold for side effects in FD patients. Additionally, lithium has been associated with an increased seizure risk, and neuroleptics may worsen extrapyramidal symptoms.

Effective therapies to control the progression of brain calcification have been rarely reported. Bisphosphonates have been reported to improve symptoms in isolated cases, although a demonstrated reduction in calcium deposition remains lacking [15].

## Conclusions

As FD exhibits clinical heterogeneity, the diagnosis relies on neuroimaging when no alternative explanation for bilateral and symmetrical basal ganglia calcifications is evident. Treatment is tailored to symptom control for FD and correction of underlying metabolic abnormalities. 

The cases presented intend to highlight the clinical and diagnostic methods and effective treatment of FD. FD is often unfamiliar to non-psychiatrists and non-neurologists, potentially leading to misdiagnosis, underdiagnosis, and inadequate treatment for affected patients. Increasing access to this information among physicians and students is essential for prompt diagnosis, enhancing the quality of care for these patients and reducing morbidity.
